# Inhibition of tumor growth in vivo by a nanoparticle-based therapeutic cancer vaccine targeting HAAH

**DOI:** 10.1186/2051-1426-1-S1-P269

**Published:** 2013-11-07

**Authors:** Steven Fuller, Solomon Stewart, Michael Lebowitz, Kanam Malhotra, Hossein Ghanbari

**Affiliations:** 1 209 Perry Parkway, Suite 13, Panacea Pharmaceuticals, Inc., Gaithersburg, MD, USA

## 

We have designed, developed and produced a lambda-phage based therapeutic anti-cancer vaccine (nanoparticle) targeting human aspartyl (asparaginyl) β-hydroxylase (HAAH). This protein is over-expressed on the surface of cancer cells and plays a central role in cancer etiology that affects cancer cell growth, motility and invasiveness. To overcome the self-antigen tolerance of the molecule, we have designed a vaccine entity that contains an immunostimulant and presents HAAH in a manner that is unfamiliar to the body. We have expressed three portions of the HAAH protein, sequences from the N-terminus, middle portion and C-terminus, as fusion proteins (with the gpD bacteriophage antigen) on the surface of bacteriophage lambda, generating 200-300 copies per phage. All three entities display highly significant, dose-dependent immunogenicity as assessed by antibody ELISAs. To evaluate the therapeutic effect of the nanoparticle vaccine, we initiated tumor formation in BALB/c mice using a mouse hepatocellulular carcinoma cell line, BNLT3. This cell line, a highly tumorigenic subclone of the ATCC cell line, BNL 1ME A.7R.1, was developed by J. Wands at the Liver Research Center, Rhode Island Hospital by three serial subcutaneous passages of the parental cell line. On Day 0 of the tumor challenge study, 4 groups of 5 mice each were administered 5E03 BNLT3 cells subcutaneously. On the same day, the animals received the first of three weekly subcutaneous doses (i.e., Days 0, 7 and 14) of nanoparticle vaccine as monovalent vaccines of each of the three bacteriophage constructs (10E10 pfu/dose) or a buffer control. The mice were then observed for tumor growth and tumor volume was determined. After 4 weeks (Study Day 28), 3 of 5 mice in the control group had measurable tumor growth, while in the HAAH N-terminus, middle portion and C-terminus construct vaccine groups, 0/5, 2/5 and 0/5 animals, respectively, had measurable tumor growth. The mean tumor volumes of the 5 animals in each of the two groups with tumor growth were 85.8 mm3 for the control group and 24.9 mm3 (29 % of control tumor volume) for the HAAH middle portion group. Overall, the vaccinated groups had growth in 2/15 animals and mean tumor growth of 8.3 mm3 (> 10 % of the control group tumor size). We are continuing to evaluate the HAAH nanoparticle vaccines in the mouse tumor model under various conditions and combinations of constructs. We are also assessing study samples for cell-based immunity as we attempt to elicit the mechanism of action for this vaccine candidate. We are also taking all the required steps in preparation for an IND submission to FDA.

